# Proactive patient safety: enhancing hospital readiness through simulation-based clinical systems testing and healthcare failure mode and effect analysis

**DOI:** 10.1186/s41077-024-00298-z

**Published:** 2024-06-26

**Authors:** Tarek Hazwani, Heba Hamam, Angela Caswell, Azza Madkhaly, Saif Al Saif, Zahra Al Hassan, Reem Al Sweilem, Asma Arabi

**Affiliations:** 1 Department of Pediatrics, Ministry of National Guard - Health Affairs, P.O. Box 22490, 11426 Riyadh, Saudi Arabia; 2 College of Medicine, King Saud Bin Abdulaziz University for Health Sciences, Riyadh, Saudi Arabia; 3 King Abdullah International Medical Research Center, Riyadh, Saudi Arabia; 4 Riyadh Clinical Simulation Center, King Saud Bin Abdulaziz University for Health Sciences, Riyadh, Saudi Arabia; 5 Department of Obstetrics and Gynecology, Ministry of National Guard - Health Affairs, Riyadh, Saudi Arabia; 6 Nursing Service, Ministry of National Guard - Health Affairs, Riyadh, Saudi Arabia; 7 Department of Neonatology, Ministry of National Guard - Health Affairs, Riyadh, Saudi Arabia

**Keywords:** Simulation, Patient safety, Hospital readiness, System testing

## Abstract

**Background:**

Recognizing and identifying latent safety threats (LSTs) before patient care commences is crucial, aiding leaders in ensuring hospital readiness and extending its impact beyond patient safety alone. This study evaluated the effectiveness of a combination of Simulation-based Clinical Systems Testing (SbCST) with Healthcare Failure Mode and Effect Analysis (HFMEA) with regard to mitigating LSTs within a newly constructed hospital.

**Methods:**

Three phases of the combined SbCST and HFMEA approach were implemented across all hospital settings. The scenarios tested system functionalities, team responses, and resource availability. The threats thus identified were categorized into system-related issues, human issues, and resource issues, after which they were prioritized and addressed using mitigation strategies. Reassessment confirmed the effectiveness of these strategies before hospital commissioning.

**Results:**

More than 76% of the LSTs were mitigated through the combined approach. System-related issues, such as nonfunctional communication devices and faulty elevators, were addressed by leadership. Human issues such as miscommunication and nonadherence to hospital policy led to improvements in interprofessional communication and teamwork. Resource issues, including missing equipment and risks of oxygen explosion, were addressed through procurement, maintenance, and staff training for equipment preparation.

**Conclusion:**

The SbCST and HFMEA were highly effective with regard to proactively identifying and mitigating LSTs across all aspects of hospital preparedness. This systematic and comprehensive approach offers a valuable tool for enhancing patient safety in new healthcare facilities, thereby potentially setting a new standard for proactive hazard identification and risk management in the context of healthcare construction and commissioning.

**Supplementary Information:**

The online version contains supplementary material available at 10.1186/s41077-024-00298-z.

## Introduction

Healthcare simulation-based training has been widely recognized for its effectiveness with regard to enhancing the knowledge, skills, and attitudes of healthcare providers across a variety of disciplines [1]. Simulation-based training has a positive impact on the ability of staff to manage high-stress situations, leading to improved confidence, skills, and knowledge among participants [2–4]. Beyond the level of individual development, simulation-based approaches have also been identified as invaluable tools for assessing and optimizing the operational readiness of healthcare facilities. In the design and construction phases of modern healthcare facilities, relevant leaders are increasingly incorporating features that support safe and efficient patient and staff care, such as controlled indoor environments, optimized interior designs, and well-planned area layouts, into the infrastructure [5, 6]. However, poorly conceived layouts or architectural decisions can inadvertently compromise patient safety by introducing systemic flaws and inefficiencies that ultimately elevate the risk of latent safety threats (LSTs) [7–9]. While human factors and ergonomics (HFE) has gained recognition for its importance in healthcare quality and patient safety, existing evidence underscores its potential to enhance the quality of care and patient safety through healthcare system redesign. Numerous models exist that can provide a deeper understanding of HFE conditions and contribute to the development of frameworks aimed at addressing ongoing design challenges to improve patient safety, human factors, and work environment solutions [10, 11].

Accordingly, in situ simulation-based approaches are now being utilized postconstruction to assess and mitigate the risk of LSTs in newly built healthcare facilities before they receive patients [5, 8, 9, 12–14]. This approach has proven to be instrumental with regard to evaluating the healthcare system’s preparedness for diverse scenarios, including disasters. Studies have further demonstrated how simulation can inform the development of new disaster response protocols that are critical for managing patient triage, resource allocation, and public communication [3, 15]. In conjunction with simulation testing, Healthcare Failure Mode and Effect Analysis (HFMEA) can be employed as a systematic tool to identify, categorize, prioritize, and mitigate the risk of LSTs. Within this framework, the notion of failure modes represents the inability of hospital-based systems to perform their intended functions, while the concept of effects refers to the potential consequences of these failures [16–18]. The HFMEA assigns numerical hazard values based on a two-pronged analysis: (1) the severity of the potential impact on patients and clinical staff and (2) the probability of the failure mode occurring [19].

Despite the increasingly widespread use of in situ simulation testing, there may be a need for further attention to its applicability and specific use in evaluating hospital readiness prior to patient admission [3, 13–18, 20–25].

This research gap limits our understanding of how preopening evaluations can inform the development of solutions and optimize hospital preparedness. Therefore, this paper aims to critically evaluate the efficacy of in situ simulation testing with regard to establishing a new hospital by assessing its effectiveness with respect to recognizing, mitigating, and addressing LSTs related to demands imposed on hospital systems, patient transportation pathways, equipment and team readiness, and overall operational efficiency.

### Setting

This prospective study was conducted at Women’s Health Hospital (WHH), a leading women’s health facility located in King Abdulaziz Medical City, the largest medical complex in Saudi Arabia. WHH is the largest hospital specializing in women’s health in the Gulf area. It boasts a total capacity of 349 beds catering to adult female inpatients, along with a 96-bed neonatal intensive care unit (NICU) and intermediate care nursery (ICN). Additionally, WHH houses day treatment facilities, a 27-bed labor and delivery unit (L&D), four operating rooms (OR), and a 27-bed gynecology obstetric triage assessment and management unit (GOTAMU).

## Methods

The study spanned 14 months, from July 2022 to September 2023, and followed a two-stage approach.

Stage 1: Development and planning. This initial stage focused on establishing a multidisciplinary team, identifying testing objectives related to high-stress situations and healthcare system readiness, and designing relevant scenarios.

**Fig. 1:**
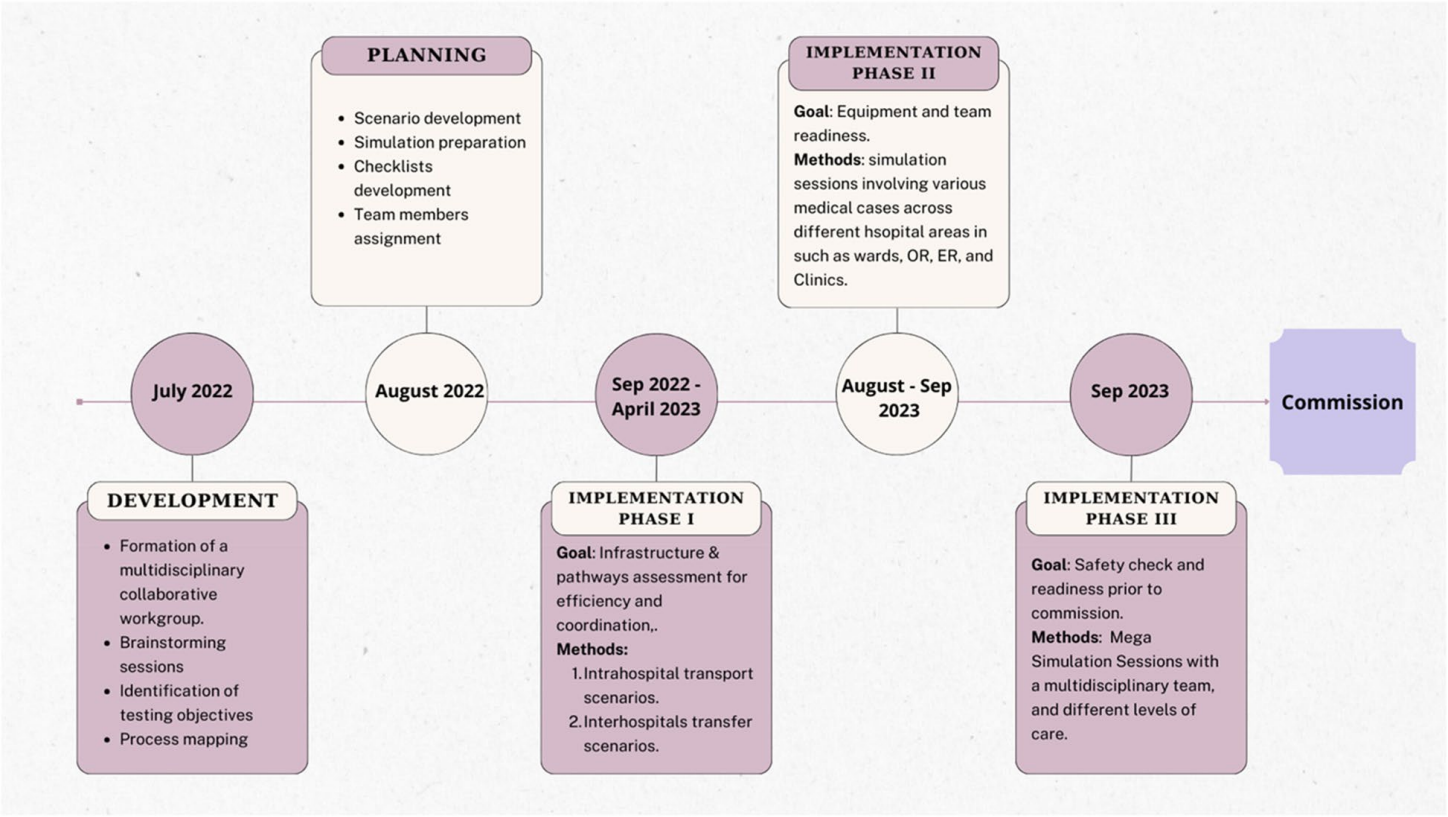
Simulation program timeline

Stage 2: Implementation. This subsequent stage encompassed three distinct phases: testing hospital infrastructure and patient transfer pathways under simulated crisis situations, evaluating equipment and team readiness through simulated crisis scenarios, and retesting the LSTs identified, along with corresponding mitigation strategies, in response to crisis situations (Fig. 1).

### Development and planning

#### The establishment of a multidisciplinary collaborative workgroup

Our simulation-based clinical systems testing (SbCST) program aimed to uncover and address LSTs that emerge before and during patient transfer to the new Women’s Health Hospital (WHH). These LSTs could pertain to the following issues:

- System and patient transportation pathways both within WHH and from the old hospital.
- Equipment readiness and functionality.
- Team preparedness and responses in high-stress situations.

To achieve this goal, we established a multidisciplinary team comprising clinical leaders, simulation experts, and frontline staff. The team collectively decided to employ the HFMA testing as an effective strategy for detecting and evaluating LSTs, aiming to enhance systems and ultimately improve patient safety [17, 26, 27].

Based on brainstorming sessions and interviews, we identified high-frequency/low-acuity (e.g., routine admissions) and low-frequency/high-acuity events that could inform subsequent steps of the process:

- Needs assessment: This step established key objectives such as identifying testing priorities, developing process maps, designing scenarios, and defining roles within the collaborative workgroup.
- Shared mental model: The cultivation of a clear understanding of the purpose and goals of the scenarios among all participants (including stakeholders unfamiliar with SbCST) was crucial for ensuring accurate analysis and open communication.
- Testing objectives: Clear communication and repetition were emphasized when conveying objectives and roles to stakeholders, clinical leaders, and participants, thus maximizing understanding and engagement. This process involved leveraging routine patient movements to assess adherence to safe design principles, such as minimizing interruptions, reducing excessive walking, and optimizing equipment placement [11].

#### Execution team

The Paediatric Simulation Training & Education Program (PediSTEP) team, working alongside clinical leaders from the obstetrics and gynecology (Ob-Gyn) department, NICU, and nursing services, formed the execution team. These participants planned and executed the program collaboratively; reviewed the proposed scenarios for feasibility, validity, and reliability, and assigned team members from relevant medical and allied services.

#### Process mapping

Detailed process maps outlining each event sequence were constructed through collaboration between team members and frontline staff. Every activity, decision point, set of personnel, supply source, set of equipment, and participant role was clearly identified for each clinical situation, process, or workflow scheduled for testing (Fig. 1).

#### *Comprehensive *in situ* testing*

The simulations encompassed both clinical and nonclinical areas (patient care, public spaces, support services, administration) to ensure a comprehensive and realistic evaluation before patient arrival. This holistic approach facilitated the functional assessment, system process evaluation, and early identification of potential patient safety concerns, thus offering more benefits than testing focused on limited hospital settings.

#### Scenario development

In collaboration with clinicians from each area, the execution team developed in situ simulation sessions focusing on routine clinical practice and crisis situations that were relevant to specific patient populations. This partnership ensured both clinical fidelity and alignment with the SbCST objectives. Simulationists anchored each task to a safe design principle, thus facilitating the robust testing of multiple elements within each scenario. The process of prioritization focused on frequent, urgent, challenging, new, or high-risk situations. Clinical complexity and complex medical decision-making were minimized to maintain a focus on system- and process-related aspects. The number and length of the scenarios depended on the scope of the evaluation, the new areas under investigation, and the distinct clinical departments involved. Individual scenario complexity influenced the duration, participant numbers, observer requirements, and debriefing length of the process.

#### Simulation preparation

During the week prior to each SbCST, simulationists meticulously assembled event materials (rosters, scripts, checklists, guides, etc.). Clinical leaders ensured that team members who were going to participate in the scenarios were notified and received confirmation, thereby extending communication to relevant services and operational units to guarantee comprehensive awareness and coordination.

### Implementation phases

The implementation stage involved three distinct phases, each of which had a specific goal:

- Phase I: Infrastructure and transfer pathways: This phase involved testing the functionality of hospital infrastructure and the effectiveness of patient transfer pathways both within WHH and between the old hospital and WHH.
- Phase II: Equipment and team readiness: This phase focused on evaluating the preparedness of equipment and team responses under simulated high-stress situations.
- Phase III: LST retesting and mitigation: This phase involved retesting previous LSTs and evaluating the effectiveness of the implementation of mitigation strategies drawn from phases I and II.

#### Simulation pre-briefing and orientation

Before each simulation session, a pre-briefing was used to establish the scenario objectives and ensure the overall flow of the process. Notably, this pre-briefing focused on the task of ensuring a psychologically safe environment in which individual performance evaluation was not the main area of focus. This approach aimed to encourage open and honest discussion, which is particularly valuable for stakeholders and leaders. Following the prebriefing, participants familiarized themselves with the space, while observers strategically positioned themselves in designated locations. The observers comprised the nursing manager, simulationists, project management office members, department managers, and clinical department chairpersons from the relevant units. They actively engaged in the debriefing session, bringing their notes and comments for discussion.

#### Data collection through debriefing

Multidisciplinary teams of frontline staff implemented care scenarios in each clinical area under examination. Structured and facilitated debriefing sessions were used to identify latent conditions and potential active failures related to the architectural design. Simulation team members meticulously transcribed these sessions into a predefined template (Additional file 1) [17]. The simulationist participated actively in this process, inputting information into a preformatted reporting template to facilitate the comprehensive documentation of the identified issues. Detailed notes were kept to outline each potential safety threat or system inefficiency alongside the corresponding potential impact on patient safety, workflow, or process efficiency. For each scenario, one or two simulationists independently completed checklists to ensure a thorough evaluation. In the event of any disagreement among simulationists, the matter would undergo further review by the simulation program chair before finalizing the report.

#### Debriefing techniques

Immediate debriefing sessions followed each simulation; these sessions were based on a standardized approach that involved scoring checklists (Additional files 1 and 2). Importantly, SbCST debriefing techniques differ significantly from traditional, education-based debriefing techniques. The SbCST approach is facilitator-focused; the facilitator from the simulation team of PediSTEP prompts reactions for each scenario step [28]. This approach guides the group in identifying safety threats and their potential impacts on patient/staff safety, workflow, process efficiency, and equipment/technology functionality [17, 28, 29]. This focused exploration ensures an in-depth evaluation of aspects that are crucial for assessing clinical scenarios in the context of hospital design.

### Data analysis

#### Hazard analysis and HFMEA scoring

Following each simulation event, a dedicated scoring group is convened to conduct HFMEA. This group comprises departmental and service line leaders, institutional operational leaders, and executive leaders, thereby representing a multidisciplinary perspective that can be used to evaluate potential risks. The HFMEA serves as a proactive risk assessment tool, guiding the team in analyzing healthcare processes. High-risk LSTs refer to incidents for which the risk priority number (RPN) exceeds 32 (Additional file 2) [17].

The simulationists utilized the HFMEA to facilitate the scoring process. They reviewed, evaluated, and scored each potential LST identified during the debriefing sessions. Each LST underwent a meticulous assessment, in which it was assigned scores for occurrence, detection, and severity. These scores then informed categorization based on system-, human-, and resource-related issues.

Following the scoring process, a comprehensive report was generated, which detailed each LST alongside its corresponding score and RPN categorization. This report was distributed to stakeholders, clinical leaders, and the quality team, thus ensuring transparent communication and proactive mitigation of potential risks identified during the simulation events.

#### Ethical statement

Ethics approval for the study was obtained from our local Institutional Review Board (IRB) prior to data collection (RO/WE/WS/008/2024). 

#### Scenario scope and risk analysis of the SbCST

The SbCST spanned three phases, including 34 scenarios extending across various hospital services, including neonatal intensive care units, GOTAMU, operating rooms, wards, and outpatient clinics (Table 1).

- Phase I: Infrastructure and transfer pathways (16 scenarios): This phase involved assessing hospital infrastructure functionality and coordination with a focus on patient transfers both within WHH and between old and new facilities.
- Phase II: Equipment and team readiness (12 scenarios): This phase focused on evaluating the preparedness of equipment and team responses under simulated high-stress situations.
- Phase III: LST retesting and mitigation (6 scenarios): This phase entailed reassessing scenarios drawn from phases 1 and 2 for safety and readiness before hospital commissioning.

**Table 1:** Characteristics of scenario themes in each phase of the simulation program

Scenario number		Theme of the scenario	Involved teams*	Area
Phase I	1	Interhospital transfer of neonatal patient from NICU KAMC to WHH by EMS	NICU, nursing, EMS, RT, military	NICU
	2	Intrahospital transfer of the neonatal critical patient from NICU KAMC to WHH by EMS	NICU, nursing, EMS, RT, military	NICU
	3	Triage of preeclampsia patient	Ob-Gyn, ER, nursing, PMO	GOTAMU
	4	Interhospital transfer of patient from OR to PACU, then to ward	OR, PACU, PMO	OR
	5	Intrahospital transportation of patients for elective C/section	Ob-Gyn, nursing, NICU, PMO	Ward, L&D OR
	6	Intrahospital transportation of antenatal patient in active labor to L&D	Ob-Gyn, nursing, NICU, PMO	Ward, L&D OR
	7	Intrahospital transportation of newborn baby from L&D OR to NHDU	NICU, nursing, PMO	L&D OR, NHDU
	8	Intrahospital transportation of Ob-Gyn patient with tachycardia to ER	Nursing, Ob-Gyn, PMO	Outpatient clinic
	9	Intrahospital transportation of outpatients to L&D	Nursing, Ob-Gyn, PMO	Outpatient clinic
	10	Interhospital coaster transfer of stable postpartum mothers from KAMC to WHH	Ob-Gyn, nursing, military	Ward
	11	Interhospital coaster transportation of sick postpartum mothers from KAMC to WHH	Ob-Gyn, nursing, military, EMS, ER	Ward
	12	Intrahospital transportation of CCRT patients from ward to ICU KASCH	Ob-Gyn, nursing, ICU, CCRT, RT, pharmacy	Ward, ICU
	13	Intrahospital transportation of CCRT patient from GOTAMU to ICU KASCH	ICU, Nursing, RT, Pharmacy, GOTAMU	GOTAMU, ICU
	14	Interhospital transfer of stable neonatal patient from ICN to WHH	Nursing, NICU, RT, clinic, military	ICN, NHDU
	15	Interhospital transfer of unstable neonatal patient from ICN to WHH	Nursing, NICU, RT, clinic, military	ICN, Clinic, NHDU
	16	Code Pink	Nursing, NICU, military	NHDU
Phase II	1	Triage level 1 patient collapsed in the third trimester, code blue	Nursing, Ob-Gyn, CCRT, code blue, anesthesia, RT, pharmacy, social services	GOTAMU
	2	Intrahospital transfer of a patient in active labor to the delivery suite	Nursing, L&R, Ob-Gyn, NICU, Lab	GOTAMU, L&D
	3	Code white activation with a violent husband, threatening staff with violence	Nursing, military, patient experience, social services, Ob-Gyn	L&D
	4	A 45-year patient post-abdominal hysterectomy day one postoperative. Estimated blood loss 250 ml. Reported chest discomfort, followed by a considerable hemorrhage requiring OR	Nursing, OR, Ob-Gyn, anesthesia, pharmacy, medical imaging	L&D
	5	Rapid response and transfer of the patient from level 5 to L&D OR	Nursing, OR, L&D, Ob-Gyn, NICU, CCRT, anesthesia, RT, Lab, KASCH	Ward, OR
	6	CCRT activation progressing to code blue. Transfer patient to ICU C55 in KASCH	Nursing, Ob-Gyn, CCRT, code blue, RT, pharmacy, social services	Ward
	7	Neonatal code blue activation in SCBU	Nursing, NICU, RT, pharmacy, social services	Non Clinical area
	8	Intrahospital transfer of neonate from NICU to emergency OR	NICU, KASCH, Anesthesia, RT	NICU
	9	Code pink activation of a suspected abducted infant	Nursing, NICU, military, patient experience, social services	L&D
	10	Transfer of patient from GOTAMU to OR	NICU, ER, OR, anesthesia, lab, nursing, Ob-Gyn	GUTAMO&OR
	11	Intrahospital transfer of Ob-Gyn booked patient with tachycardia to ER	Nursing, ER, Ob-Gyn	ACC & GUTAMO
	12	ACC patient journey	Nursing	ACC
Phase III	1	Code blue, nonclinical area	EMS	Nonclinical area
	2	Transfer of patient from W76 to OR for emergency C/section	Nursing, OR, Ob-Gyn, anesthesia, lab	Ward, OR
	3	Elective C/S, difficult intubation, urology consultation	OR, nursing, anesthesia, lab	OR
	4	Patient bleed required interventional radiology Transfer patient from PACU to ANGIO	Nursing, Ob-Gyn, Lab, KASCH	PACU
	5	Code blue in discharge lounge	Nursing, ER, Ob-Gyn, code blue, RT, pharmacy, social services	Discharge lounge
	6	Code blue in discharge lounge	Nursing, ER, Ob-Gyn, code blue, RT, pharmacy, social services	Discharge lounge

^*^Involved teams include physicians, nurses, and workers from the mentioned units

*NICU* neonatal intensive care unit, *KAMC* King Abdulaziz Medical City, *WHH* Women’s Health Hospital, *EMS* emergency medical services, RT respiratory therapist, *ER* emergency room, *Ob-Gyn*, obstetrics and gynecology, *GOTAMU* Gynecology Obstetric Triage Assessment and Management Unit, *PMO* project management office, *PACU* post-anesthesia care unit, *OR* operation room, *L&D* labor and delivery, *NHDU* neonatal high dependency unit, *CCRT* critical care response team, *ICN* intermediate care nursery, *SCBU* special care baby unit, *ACC* ambulatory care clinics, *ICU* intensive care unit, *KASCH* King Abdullah Specialized Children’s Hospital, *Code blue* cardiopulmonary arrest code announcement, *Code pink* missed baby code announcement, *Code white* violence event announcement

The scenarios involved the participation of multidisciplinary teams such as military and nursing staff, obstetrics and gynecology teams, neonatal intensive care units, anesthesia units, laboratories, and social care units (Table 1).

### Outcomes of the SbCST/HFMEA testing

#### Identified LSTs

A total of 136 LSTs were identified through HFMEA risk analysis, in which context phases I and II revealed more LSTs (94) than did phase III (24). Of these LSTs, 97 were considered to be high risk (RPN > 32), and 39 were considered to be low risk (RPN < 32). The number of high-risk threats per scenario varied from 0 to 7, with scenarios 7 and 10 in phase 1 and scenario 11 in phase 2 representing the extremes (Table 2). The highest threats thus detected occurred in phase I and phase II rather than in phase III. Following the application of phase I and II testing, action plans were derived for the mitigation of the risks thus detected (Table 3). These action plans, which contained recommended actions and changes, were submitted to a leadership panel to allow the recommended changes to be implemented.

**Table 2:** Detected LSTs per RPN

	**SCENARIO NO**	**TOTAL DETECTED RPN**	**RPN > 32**	**RPN < 32**
**PHASE I**	1	3	2	1
	2	2	2	0
	3	6	3	3
	4	5	2	3
	5	3	1	2
	6	6	4	2
	7	7	3	4
	8	5	3	2
	9	5	2	3
	10	7	5	2
	11	3	3	0
	12	5	4	1
	13	3	3	0
	14	1	1	0
	15	6	6	0
	16	3	2	1
**PHASE II**	1	1	1	0
	2	5	1	4
	3	6	5	1
	4	5	4	1
	5	5	5	0
	6	7	5	2
	7	2	2	0
	8	1	0	1
	9	6	5	1
	10	3	3	0
	11	0	0	0
	12	1	0	1
**PHASE III**	1	2	2	0
	2	3	2	1
	3	3	3	0
	4	5	4	1
	5	6	5	1
	6	5	4	1

*RPN* risk priority number

**Table 3:** Action plan for LSTs per RPN > 32

Scenario no	RPN > 32	LST category	Potential failure effect	Recommended actions	Change made by leadership
1	Not all phones were working	System issue	Difficulty and delay in communication	Ensure all phones are working in all areas	Immediate action is required to install, connect, and test all phones
2	CTG machine, portable Doppler, O_2_ tank, and medications were not available	Resource issue	Patient intervention and treatment could not be rendered	Ensure all essential equipment and supplies are prepared in the rooms	All essential equipment and supplies are in place
3	Coding system not functioning	System issue	Difficulty in code announcements	Contact communication service	Emergency Code System tested
3	Coding system is not available on all desktop computers	System issue	Difficulty in code announcements and relying on human factor by calling 911	Contact communication service to ensure all desktop computers and laptops have the Coding system icon	Matter resolved
3	Military police pagers are not configured to all coding systems	System issue	Delayed response to codes	Ensure all military police pagers are configured to receive the codes that require their presence	Matter resolved
3	Command center 911 activated code white after 25 min	System issue/human factors	Delayed response to codes and possible escalation of violent behavior	Contact disaster management for possible solutions	Matter resolved
3	Command center 911 did not announce code white correctly	System issue/human factors	Delayed team response due to unclear announcement	Contact disaster management for possible solutions	Matter resolved
4	Unavailability of anesthesia and medical imaging teams	Human factor	Patient treatment and intervention could not be rendered	Communicate the schedule of simulations to all the team and confirm their attendance	Matter resolved
4	Portable U/S machine was not available	Resource issue	Patient’s condition could not be safely assessed	Ensure all the equipment is ready	Matter resolved
4	No emergency transport box	Resource issue	Patient could not be transferred safely	Ensure the patient is transported safely	Matter resolved
4	Trauma elevator was closed, and the other patient elevators couldn’t be accessed by the staff access cards	System issue	Patient could not be transferred safely	Communicate with the concerned departments	Decision made to enable all elevators and no access cards
5	Anesthesia was not available for the session	Human factors	Inability to arrange emergency OR booking	Ensure all team members are aware of the simulation	Matter resolved
5	Coding system not functioning	System issue	Difficulty in code announcement	Contact communication service	Matter resolved
5	The CCRT announcement was not clear, very low tone, not heard in office areas	Human factors	Delayed response from the team	Contact disaster management for possible solutions	Matter resolved
5	No notification was received on the CCRT pager	System issue	Difficult to retrieve patient information and order treatment during emergency	Communicate with informatics service	Matter resolved
5	HIS not working in the laptops	System issue	Difficult to retrieve patient data and put orders	Communicate with informatics service	Matter resolved
6	Anesthesia was not available for the session	Human factors	Inability to arrange emergency OR booking	Ensure all team members are aware of the simulation	Matter resolved
6	Coding system not functioning	System issue	Difficulty in code announcement	Contact communication service	Matter resolved
6	The CCRT and code blue announcement was not clear, very low tone, not heard in office areas	Human factors	Delayed response from the team	Contact disaster management for possible solutions	Matter resolved
6	No notification was received on the CCRT and code pagers	System issue	Delayed response from the team	Contact communication service	Matter resolved
6	The holder for the oxygen tank was small, the tank was placed horizontally on the bed	Resource issue	High risk for leak or damage	Arrange for holders that fit the available oxygen cylinders	Matter resolved
7	Coding system not functioning	System issue	Difficulty in code announcement	Contact communication service	Matter resolved
7	Neonatal code blue announcement was not clear, very low tone, not heard in office areas	Human factors	Delayed response from the team	Contact disaster management for possible solutions	Matter resolved
9	Not all phones are working	System issue	Delayed response and management	Ensure all phones are in working order	All phones in every area were tested and working
9	Coding system not functioning	System issue	Difficulty in code announcement	Contact communication service	Matter resolved
9	Command center 911 was called but the code pink announcement was delayed and incorrect	System issue/human factors	Delayed response from the team	Contact disaster management for possible solutions	Matter resolved
9	Military police did not respond to the unit of the missing infant	Human factors	No adherence to the policy and lack of communication	Reiterate the roles and responsibilities for all responders as stated in the policy	Matter resolved
9	The last seen location of the Hugs tags (Infant security system) was not communicated to military police	Human factors	Delay in locating the missing infant	Reiterate the roles and responsibilities for all responders as stated in the policy	Matter resolved
10	Not all phones are working	System issue	Delayed response and management	Ensure all phones are in working order	Matter resolved
10	HIS was not working on all computers	System issue	Difficulty in retrieving patient information and ordering treatment	Communicate with informatics service	Matter resolved
10	OR was not ready to receive patients and no equipment was available	Resource issue/Human factor	Unable to perform surgery or secure airway for the patient in OR	Communicate with the anesthesia department to ensure the OR readiness	Matter resolved

*CTG* cardiotocography, *OR* operation room, *CCRT* critical care response team, *HIS* health information system, *Code blue* cardiopulmonary arrest code announcement, *Code pink* missed baby code announcement, *Code white* violence event announcement

#### LST categorization and action plans

Based on the risk analysis, action plans were developed to address potential LSTs (Table 3). LSTs were further categorized into three groups:

- System-related issues: Unclear/low-tone code announcements, malfunctioning equipment (critical care response team (CCRT), pager, phones, computers, Health Information System (HIS)), inaccessible elevators, and lack of policy adherence and understanding of responsibilities, which is a major human factor, led to system-related failures such as improper code announcement. These failures significantly impacted the response sequence system.
- Human issues: Delays in code activation (2–25 min), incorrect/incomplete information from code agents, unavailable teams (such as anesthesia or imaging teams), the lack of prepared operating rooms, and miscommunication among responders contributed to human factor-related failures.
- Resource issues: The lack of available resources such as cardiotocography (CTG) machines, ultrasound machines, portable Doppler devices, oxygen tanks, personal protective equipment (PPEs), medications, operating room equipment, and emergency transport boxes containing essential emergency equipment required during patient transfer, alongside inadequate oxygen holder design, were categorized as resource-related failures. To differentiate between issues related to availability and those due to the hospital not yet being operational, we considered the timing of the simulations. As phases II and III were conducted before the hospital’s opening, any unavailable resources during this period were attributed to resource-related failures rather than issues arising from the hospital not being operational.

## Discussion

The combination of SbCST along with HFMEA assessment effectively identifies and categorizes LSTs. This approach aids in exploring potential risks and prioritizing their resolution before they impact patients negatively. Detection of hazards and risks facilitates the prioritization of the process of addressing and retesting before patients are negatively impacted [30]. This study aimed to use in situ simulation testing processes alongside HFMEA tools to recognize, mitigate, and address LST-imposed demands on hospital systems, transportation processes, equipment, and team readiness. The outcomes of the three phases involved in the SbCST and HFMEA approaches enabled threats to be categorized into three areas: (1) system-related issues, (2) human issues, and (3) resource issues. The findings concerning phase III showed that the LSTs decreased by 76%, thus suggesting two conclusions: (1) the risk mitigation solutions were effective in some cases, and (2) the actions taken did not solve each problem; thus, additional modifications were needed. Leadership was able to correct 100% of the LSTs thus identified before the commencement of patient admissions to the hospital.

All system-related risks identified, including delays in code announcements, elevator failures, errors in the paging system, malfunctions in phones and computers, and errors in the HIS, were addressed by leadership (Table 3). These system-related issues can lead to various negative consequences, underscoring the urgency of action. For instance, the malfunctioning code activation system was noted in multiple clinical areas, prompting a special meeting and discussion with the communication department to address this issue.

Similarly, all human-related LSTs that were included in this study were addressed by leaders (Table 3). The LSTs thus identified included delayed and/or absent code activation and team response for two reasons. First, miscommunication between healthcare individuals may have occurred, such as situations in which incomplete or incorrect pink code (missing baby) information was provided. Such failure modes could result in a delay in the location of missing infants by military police. A link between ineffective interprofessional communication and poor patient outcomes has previously been documented [31, 32]. Foronda et al. reported that interprofessional miscommunication can lead to delayed patient treatment, patient dissatisfaction, medical errors, and death [33].

All LSTs pertaining to resources were also addressed by leadership. Between 39 and 85% of documented cases of patient safety hazards were related to equipment and supplies [13, 34]. In this study, some required equipment and supplies were not available. For example, a lack of operating room equipment could prevent care teams from performing surgery or securing patients’ airways in the operating room. Additionally, unavailable emergency transport boxes could result in a failure to manage the risks associated with patient transfers.

Our findings underscored the importance of HFMEA testing in identifying and addressing LSTs, ultimately improving patient safety. Further research and reporting into the success of SbCST is essential to understand its effectiveness and promote its wider adoption, fostering a healthcare environment where patient safety is integral to care delivery. Additionally, it aids leaders in ensuring hospital readiness, extending its impact beyond patient safety alone.

### Strengths and limitations

This study has three major strengths. First, conducting the work prior to hospital commissioning, thereby avoiding the bustling patient environment, is a noteworthy advantage. Second, the study employed a rigorous methodology to identify, categorize, prioritize, and mitigate the included LSTs by using in situ simulation in conjunction with the HFMEA tool and group debriefings. This approach may facilitate the reporting of credible findings and ensure that trustworthiness, validity, and reliability are maintained. Third, the reassessment phase allowed us to retest and determine the effectiveness of the mitigation strategies before hospital commissioning. However, it is important to acknowledge the limited generalizability of simulation findings to other institutions due to the inherent differences between healthcare facilities.

This study is subject to several limitations. First, the simulation program was conducted after the completion of the hospital infrastructure, thereby limiting its impact on major design changes in the hospital. Early engagement of the hospital architectural team is crucial in the schematic design evaluation process to address this limitation effectively. Second, while the simulation program focused on hospital readiness, team response, equipment, and patient transfer, it did not encompass other crucial components of healthcare safety such as medication safety, fall prevention, and prevention of hospital-acquired infections. Although these components may have established programs from the old hospital, they remain important safety measures to be considered in a new clinical environment. Third, the project has a large scope, and more than one year of concerted effort was required for its implementation. This process required coordination with all hospital departments, stakeholders, healthcare providers, and simulation experts, which would be challenging in other hospital settings due to the need to free the scheduled participants from their clinical duties for the duration of hospital readiness testing. Fourth, all scenarios and debriefings included in this research were aimed at testing hospital readiness rather than staff competence. This research may thus not be relevant for researchers aiming to evaluate and improve health team competencies. Thus, further healthcare simulation research is needed to investigate training with regard to assessing and improving team readiness prior to the provision of patient services. Fifth, the presence of senior observers may have induced stress among participants and influenced their behavior. However, during the pre-briefing sessions, we emphasized to participants that the primary goal of these simulations was to assess system functionality and ensure safety measures, rather than to evaluate individual skills or knowledge. We believe that by emphasizing this point, we minimized any potential impact on participants’ performance due to the observers’ presence. To mitigate this effect further, the use of video cameras for remote observation could be considered.

## Conclusion

SbCST and HFMEA represent powerful and proactive approaches to patient safety, as demonstrated by this study and supporting research on this topic. They facilitate the detection and resolution of LSTs before the hospital’s actual commissioning. By addressing the limitations of traditional methods and embracing continuous improvement, this approach offers immense potential to establish safer healthcare environments for both patients and providers.

### Supplementary Information

Supplementary Material 1.

Supplementary Material 2.

## Data Availability

The data underlying this article will be shared on reasonable request to the corresponding author.

## Abbreviations

LSTs: Latent szafety threats
SbCST: Simulation-based Clinical Systems Testing
HFMEA: Healthcare Failure Mode and Effect Analysis
WHH: Women’s Health Hospital
NICU: Neonatal intensive care unit
ICN: Intermediate care nursery
L&D: Labor and delivery
OR: Operating room
GOTAMO: Gynecology Obstetric Triage Assessment and Management Unit
PediSTEP: Pediatric Simulation Training & Education Program
Ob-Gyn: Obstetrics and gynecology
RPN: Risk priority number
CCRT: Critical care response team
HIS: Health information system
CTG: Cardiotocography
