# Supplementary material for: Proactive patient safety: enhancing hospital readiness through simulation-based clinical systems testing and healthcare failure mode and effect analysis
Source: Adv Simul (Lond). 2024 Jun 26;9:26. doi: 10.1186/s41077-024-00298-z (PMC11202391; doi:10.1186/s41077-024-00298-z)
Supplement: Supplementary file 1 — Supplementary Material 1. [file 41077_2024_298_MOESM1_ESM.pdf]

Pedi STEP
PEDIATRIC SIMULATION TRAINING & EDUCATION PROGRAM

Appendix A: Focused debriefing guide

**Phase**	**Explanation**	**Comments**
**Summarize**	Facilitator reviews goals of the debriefing and summarize the scenario	•<br>•<br>•<br>•
**Anchor the clinical context**	Facilitator orient team to each phase of care within the scenario in chronological order	•<br>•<br>•<br>•
**Facilitate identification of latent threat**	Facilitator asks question related to latent threats	•<br>•<br>•<br>•
**Explore potential active failure**	Facilitator elicits impact of latent threats	•<br>•<br>•<br>•
**Elicit additional feedback**	Facilitator elicits feedback from the observers and other team members.	•<br>•<br>•<br>•

Adapted from: SAFEE: A Debriefing Tool to Identify Latent Conditions in Simulation-based Hospital Design Testing, Advances in Simulation (2020)

Simulation goals category:

**System-related issues**	**Human issues**	**Resource issues**
