# Supplementary material for: Proactive patient safety: enhancing hospital readiness through simulation-based clinical systems testing and healthcare failure mode and effect analysis
Source: Adv Simul (Lond). 2024 Jun 26;9:26. doi: 10.1186/s41077-024-00298-z (PMC11202391; doi:10.1186/s41077-024-00298-z)
Supplement: Supplementary file 2 — Supplementary Material 2. [file 41077_2024_298_MOESM2_ESM.pdf]

HFMEA scoring guide

Healthcare Failure Mode and Effect Analysis (HFMEA) SCORING TOOL				
	**4**	**3**	**2**	**1**
**SEVERITY**	**CATASTROPHIC**<br>*Failure could cause death, injury*	**MAJOR**<br>*Failure could cause high degree customer dissatisfaction*	**MODERATE**<br>*Failure can be overcome, but there is minor performance loss*	**MINOR**<br>*Failure not noticeable to customer, no effect on delivery of service*
**PROBABILITY OR OCCURRENCE**	**FREQUENT**<br>*Likely to occur immediately or within a short period (may happen several times in 1 year)*	**OCCASIONAL**<br>*Probably will occur (may happen several times in 1 to 2 years)*	**UNCOMMON**<br>*Possible to occur (may happen sometime in 2 to 5 years)*	**REMOTE**<br>*Unlikely to occur (may happen sometime in 5 to 30 years)*
**DETECTION**	**ALMOST IMPOSSIBLE**<br>*No known controls are available to detect failure mode*	**REMOTE**<br>*Remote likelihood that current controls will detect failure mode*	**MODERATE**<br>*Moderate likelihood that current controls will detect failure mode*	**HIGH**<br>*High likelihood that current controls will detect failure mode. Reliable detection controls are known with similar processes*
**Risk Priority Number (RPN)** is calculated by multiplying Severity score by Probability score by the Detection<br>*(Issues are considered significant priorities if RPN is between 32-64 on scale of 1-64)*				

Adapted from: Simulation-based clinical systems testing for healthcare spaces: from intake through implementation, Advances in Simulation (2019)

Threat Detected	LSTs Category (Latent Safety Threats)	Potential Failure Effect	Action Recommended by Scoring Team	Severity	Occurrence	Detection	RPN
What is the process step, change or feature under investigation?	*Resource Issue, Process/Systems Issue, Facility Issue, Clinical*	*What is the impact on the customer if this failure is not prevented or corrected?*	*What are the recommended actions for reducing the occurrence of the cause or improving detection?*				

HFMEA scoring guide

Scenario Number:	Theme of scenario:
Scenario date:	Area:

Threat Detected	LST Category	Potential Failure Effect	Action Recommended by Scoring Team	Severity	Occurrence	Detection	RPN
